# Retraction Note: Embelin alleviates weaned piglets intestinal inflammation and barrier dysfunction via PCAF/NF-κB signaling pathway in intestinal epithelial cells

**DOI:** 10.1186/s40104-024-01130-4

**Published:** 2024-12-06

**Authors:** Weilei Yao, Tongxin Wang, Lu Huang, Zhengxi Bao, Shu Wen, Feiruo Huang

**Affiliations:** Department of Animal Nutrition and Feed Science, College of Animal Science and Technology, Huazhong Agricultural University, Wuhan, 430070 China

**Retraction Note: J Anim Sci Biotechnol 13, 139 (2022)**

**https://doi.org/10.1186/s40104-022-00787-z**

The Editor-in-Chief has retracted this article at the request of the Huazhong Agricultural University. An institutional investigation determined that there are concerns regarding the reliability of the data presented in this work, as this data differs substantially from that reported in Dr Wen's dissertation. These concerns call into question the integrity of the data and the article's overall scientific soundness. The Editor-in-Chief therefore no longer has confidence in the research presented in this work.

The authors have not replied to correspondence from the Publisher.

